# Determinants of Sensitivity to DZNep Induced Apoptosis in Multiple Myeloma Cells

**DOI:** 10.1371/journal.pone.0021583

**Published:** 2011-06-24

**Authors:** Zhigang Xie, Chonglei Bi, Lip Lee Cheong, Shaw Cheng Liu, Gaofeng Huang, Jianbiao Zhou, Qiang Yu, Chien-Shing Chen, Wee Joo Chng

**Affiliations:** 1 Cancer Science Institute of Singapore, National University of Singapore, Singapore, Singapore; 2 Department of Medicine, National University Health System, Singapore, Singapore; 3 Department of Haematology and Oncology, National University Health System, Singapore, Singapore; 4 Molecular Pharmacology, Genome Institute of Singapore, Singapore, Singapore; 5 Division of Hematology and Oncology, School of Medicine, Loma Linda University, Loma Linda, California, United States of America; Cleveland Clinic, United States of America

## Abstract

The 3-Deazaneplanocin A (DZNep), one of S-adenosylhomocysteine (AdoHcy) hydrolase inhibitors, has shown antitumor activities in a broad range of solid tumors and acute myeloid leukemia. Here, we examined its effects on multiple myeloma (MM) cells and found that, at 500 nM, it potently inhibited growth and induced apoptosis in 2 of 8 MM cell lines. RNA from un-treated and DZNep treated cells was profiled by Affymetrix HG-U133 Plus 2.0 microarray and genes with a significant change in gene expression were determined by significance analysis of microarray (SAM) testing. *ALOX5* was the most down-regulated gene (5.8-fold) in sensitive cells and was expressed at low level in resistant cells. The results were corroborated by quantitative RT-PCR. Western-blot analysis indicated ALOX5 was highly expressed only in sensitive cell line H929 and greatly decreased upon DZNep treatment. Ectopic expression of ALOX5 reduced sensitivity to DZNep in H929 cells. Furthermore, down-regulation of *ALOX5* by RNA interference could also induce apoptosis in H929. Gene expression analysis on MM patient dataset indicated *ALOX5* expression was significantly higher in MM patients compared to normal plasma cells. We also found that Bcl-2 was overexpressed in DZNep insensitive cells, and cotreatment with DZNep and ABT-737, a Bcl-2 family inhibitor, synergistically inhibited growth and induced apoptosis of DZNep insensitive MM cells. Taken together, this study shows one of mechanisms of the DZNep efficacy on MM correlates with its ability to down-regulate the ALOX5 levels. In addition, DZNep insensitivity might be associated with overexpression of Bcl-2, and the combination of ABT-737 and DZNep could synergistically induced apoptosis. These results suggest that DZNep may be exploited therapeutically for a subset of MM.

## Introduction

The 3-Deazaneplanocin A (DZNep) is the cyclopentanyl analog of 3-deazaadenosine that inhibits the activity of S-adenosyl-L homocysteine (AdoHcy) hydrolase, the enzyme responsible for the reversible hydrolysis of AdoHcy to adenosine and homocysteine [1], [2]. Inhibition of AdoHcy hydrolase results in the cellular accumulation of AdoHcy, which leads to inhibition of S-adonosyl-L-methionine-dependent methyltransferase (MTase) activity. DZNep has been explored for antiviral treatment and has been reported to have minimal toxicity in vivo [3], [4], [5]. Recently, DZNep was reported to decrease levels of the PRC2 protein complex in breast cancer cells and inhibit associated histone H3K27Me3. It induced efficient apoptotic cell death in cancer cells but not in normal cells [6]. DZNep was also shown to deplete PRC2 complex, decrease levels of H3K27Me3, and induce apoptosis of cultured and primary acute myeloid leukemia (AML) cells. Furthermore, cotreatment with DZNep and the pan-histone deacetylase inhibitor panobinostat induced synergistic apoptosis of cultured and primary AML cells [7]. These make DZNep a possible candidate as an epigenetic therapeutic for the treatment of cancer.

Multiple myeloma (MM) is a malignant plasma cell disorder that accounts for 0.8% of all cancers worldwide[8]. Incidence rates vary from 0.4 to 5 per 100 000, and is high in North America, Australia/New Zealand, northern Europe, and western Europe compared with Asian countries [8]. Median survival for patients with MM after conventional treatments is 3–4 years; high-dose treatment followed by autologous stem-cell transplantation can extend median survival to 5–7 years [9]. As such, the development of innovative therapies and identification of more effective drugs remain high priorities for MM research. Because of its antitumor properties, DZNep holds promise as a treatment for MM. To advance DZNep as anticancer agents, it is crucial to understand the molecular mechanism and delineate markers that identify the subset of tumors that are sensitive to DZNep-induced apoptosis. In the study described here, we assayed a panel of MM cell lines and showed that DZNep at low nanomolar concentratins potently induced apoptosis in some of MM cell lines. We further investigated the mechanisms of DZNep-induced apoptosis in MM cells.

## Materials and Methods

### Cell lines and cell culture

Human MM cell lines KMS11, KMS12BM, KMS18, MM.1S, RPMI-8226, OPM-2, U266 were maintained in RPMI 1640, supplemented with 10% fetal bovine serum (FBS), 100 U/mL penicillin and 100 µg/mL streptomycin. MM cell line NCI-H929 was cultured in RPMI 1640 with 15% FCS and 0.00036% 2-mercaptoethanol. All cells were grown at 37 °C in a humidified atmosphere with 5% CO_2_. All cells were gifts from Mayo clinic, Scottsdale, AZ.

### Drugs

DZNep was kindly provided by Prof. Yu Qiang, Genome Institute of Singapore. The ABT-737, a Bcl-2 family inhibitor, was kindly provided by Prof. Chen Chien-Shing, Loma Linda University, CA. DZNep or ABT-737 was dissolved in neat DMSO (Sigma-Aldrich ) at 10 mmol/L and stored as frozen aliquots at −20°C. Caspase Inhibitor Z-VAD-FMK (Promega) was dissolved in DMSO and stored at −20°C.

### Cell proliferation assay and apoptosis analysis

Cells were seeded into 96-well plates at a density of 3.0×10^4^ cells/well in 50 µL culture medium. Then,50 µL of doubly concentrated drug solutions was added. Cells were incubated at 37°C in a humidified, 5% CO2 atmosphere for 72 h, and subsequently analyzed using the MTS colorimetric assay (Promega) as previously described [10]. The combination index (CI) for DZNep and ABT-737 combination was obtained using software CalcuSyn as described previously [11]. A CI of less than, equal to, and more than 1 indicates synergy, additivity, and antagonism, respectively. For apoptosis analysis, cells were treated with 0.5 µM DZNep and cultured for 72 h. Then, cells were pooled and analyzed for the level of apoptosis by flow cytometry using propidium iodide (PI) staining as described previously [12], [13].

### Microarray analysis and quantitative RT-PCR

Cells were treated with 0.5 µmol/L DZNep for 48 h. Total RNA was extracted by using the Qiagen RNeasy Mini kit (Germany). Gene expression was performed using the GeneChip® Human Genome U133A Array (Affymetrix) following the manufacturer's instructions. Data analysis was performed using GeneSpring software from Agilent Technologies. The data was deposited in Gene Expression Omnibus with accession no. GSE26921. The differentially expressed genes were grouped using Panther Protein Classification [14]. Using oligo(dT)_20_ primer and reverse transcriptase, cDNA was created by using the Invitrogen SuperScript III First-Strand Synthesis System. The 7300 Real-Time PCR System (Applied Biosystems, CA) was used for real-time PCR amplification according to the *Power* SYBR® Green PCR Master Mix protocol. Relative quantification of gene expression was done as described in the manual using β-Actin as an internal standard and the comparative threshold cycle method. The primers for real-time PCR were listed in **Table S1**.

### Immunoblot analysis

Cells were treated with 0.5 umol/L DZNep for 72 h. Cells were collected and lysed in RIPA buffer as described previously[15]. Equal amounts of protein were separated on SDS–polyacrylamide gels and transferred to PVDF membranes. The blots were probed with antibodies against EZH2 (#3147), Cleaved Caspase-3 (Asp175) (#9661), Caspase-8 (#9746) and Caspase-9 (#9508) from Cell Signaling Technology. Antibodies against Bcl-2 (sc-509), PARP (sc-8007), H3 (sc-10809) and β-Actin (sc-69879) were from Santa Cruz Biotechnology. The antibody against ALOX5 (#160402) was from Cayman Chemical. The antibody against H3K27Me3 (#07-449) was from Millipore. Western Blotting Luminol Reagent (sc-2048) was used for detection on film from Santa Cruz Biotechnology.

### Methylcellulose colony formation assay

Methylcellulose media (H4230, MethoCult®, StemCell Technologies) consisted of 1% methylcellulose, 30% fetal bovine serum (FBS), 100 U/mL penicillin and 100 µg/mL streptomycin, and 1% bovine serum. Cells were plated in duplicate at a density of 1,500 or 3,000 cells/mL in 0.4 mL volume in 24-well plates. Plates were incubated at 37°C, 5% CO_2_, and ≥95% humidity (provided by a water dish) for 14 days. To observe colonies, cells were stained with 0.5 mg/mL metabolizable tetrazolium salt (MTT, M2003, Sigma-Aldrich), 100 µL/well. After incubating at 37°C, 5% CO_2_ for 1 h, colonies of more than 30 cells were counted. For photomicrographs, plates were viewed with an Olimpus IX71 inverted microscope at 40X magnification. Images were acquired using a digital camera DP71 and software DP Controller.

### Analysis of *ALOX5* expression in MM patient samples

The *ALOX5* expression analysis was performed on a large gene expression dataset from the University of Arkansas Medical School that is available through the Gene Expression Omnibus. Raw expression value was obtained from GEO GSE 2658 and 5900. The expression values for probes representing *ALOX5* on the U133plus 2.0 chip were first log-transformed and median centered, and then a composite relative expression value for *ALOX5* for each of patients was obtained by taking the median expression across these probes. The expression value of 22 normal plasma cells (NPC) and 351 newly diagnosed MM was analyzed. The mean+2 standard deviation (SD) was used as the upper limit of normal expression.

### Vector construction

Sequences encoding short hairpin RNA (shRNA) were cloned into pLKO.1 vector (gift of Prof. Robert A. Weinberg, Whitehead Institute for Biomedical Research, Cambridge, MA). The shRNA against ALOX5(shALOX5) sequence: 5′ **ATCCAGCTGGTCAGAATCGAGAA** 3′. The shRNA against Bcl-2 sequences: sh#82, 5′ **CATTATAAGCTGTCGCAGAGG** 3′; sh#83, 5′ **TGTGGATGACTGAGTACCTGA** 3′. The control shRNA against luciferase (shLuc) is from Prof. Robert A Weinberg. The sequence encoding ALOX5 was amplified from NCI-H929 and cloned into pLN1 vector (derived from pLKO.1 by replacing U6 promoter with CMV promoter). All clones were verified by sequencing.

### Virus production and infection

Lentivirus infection was performed as previously described.[16] Briefly, lentiviruses were produced by transiently cotransfecting 293T cells with the shRNA-expressing lentivirus vector, packaging plasmid pCMV-dR8.2 dvpr and VSV-G envelope plasmid pCMV-VSVG (gifts of Prof. Robert A. Weinberg) using Fugene 6 (Roche Diagnostics) according to the manufacturers' instructions. Cells were infected with lentiviruses in the presence of 8 µg/mL polybrene (Sigma-Aldrich). The NCI-H929 cell line overexpressing ALOX5 was generated using puromycin (3 µg/ml, Sigma-Aldrich) selection.

### Statistical Analysis

Results were expressed as mean values ± SD, and t-test was used for evaluating statistical significance. Results were considered to be significant when *p*<0.05.

## Results

### MM cell lines display differing degree of sensitivity to DZNep

**Figure 1:**
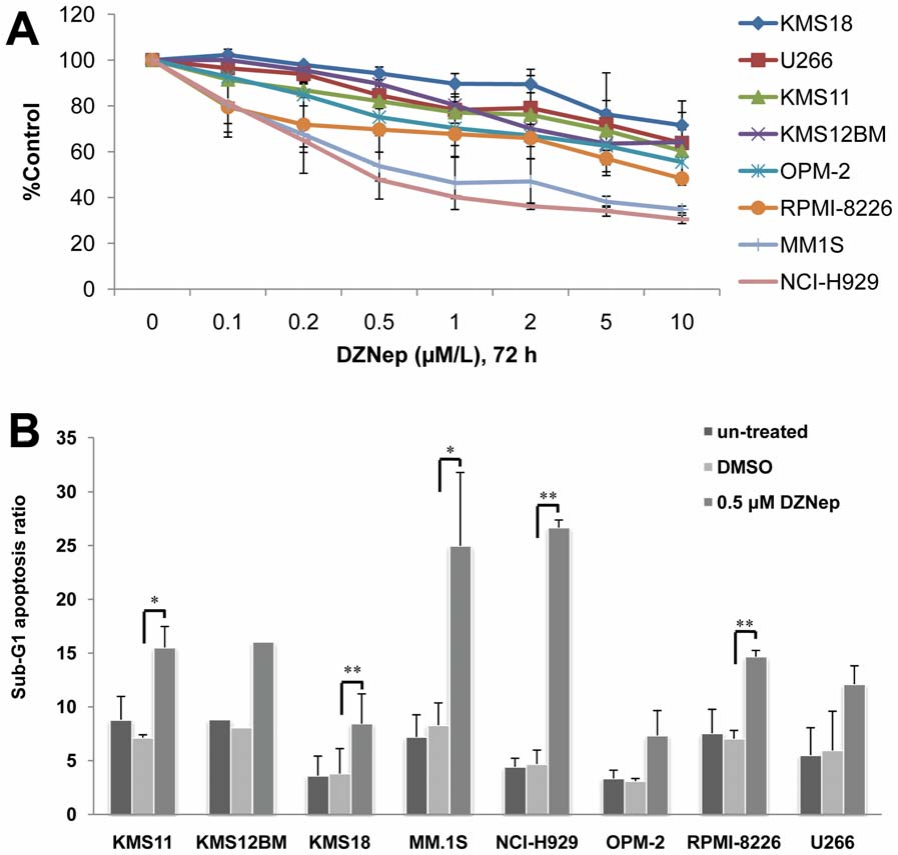
MM cell lines display differing degrees of sensitivity to DZNep. **(A)** Cells were treated with various concentrations of DZNep for 72 h. The proliferation was determined by MTS colorimetric assay (Promega). Data represent the mean ± SD derived from 3 separate experiments with triplicate wells per condition. **(B)** Cells were treated with 0.5 µM DZNep for 72 h, and apoptosis was measured by flow cytometric sub-G1 analysis. Data represent the mean ± SD derived from 3 separate experiments (one time for KMS12BM). * indicates p<0.05, and ** indicates p<0.01.

To determine the sensitivity to DZNep, cells were treated with different concentrations of DZNep, and cell proliferation was measured by MTS colorimetric assay (**Fig. 1A**). Using this approach, NCI-H929 and MM.1S, 2 of the 8 cell lines tested, were very sensitive to DZNep-induced growth inhibition in the nanomolar range. Cell apoptosis induced by DZNep was determined by subdiploid DNA content analysis using flow cytometry. Sub-G1 fraction undergoing apoptosis was significantly increased after DZNep treatment in NCI-H929 and MM.1S, while a minor to moderate increase in apoptosis was observed in the relatively insensitive cell lines. (**Fig. 1B**). Because there are MM cell lines that are both sensitive and insensitive to DZNep, we had the opportunity to examine the molecular mechanisms of sensitivity to DZNep within a similar tumor type.

### ALOX5 is differentially expressed in MM cells according to DZNep sensitivity

**Figure 2:**
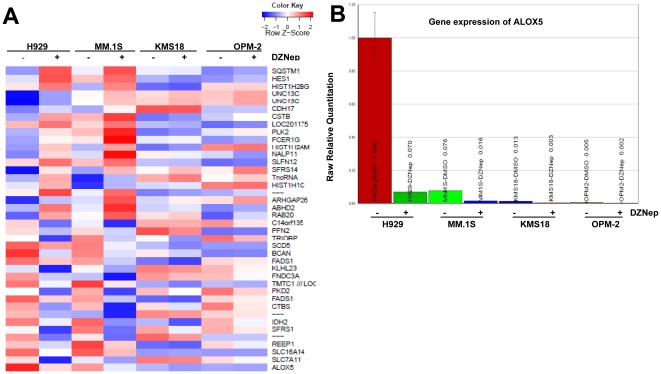
ALOX5 is differentially expressed in MM cells being sensitive to DZNep. Cells were treated with 0.5 µmol/L DZNep for 48 h. Total RNA was extracted using the Qiagen RNeasy Mini kit. **(A)** The cDNA microarray analysis was done to evaluate gene expression changes in response to DZNep treatment. Heat map showed the relative expression levels of the top 20 probe sets that were differentially expressed between DZNep-sensitive and DZNep-insensitive MM cell lines. *ALOX5* was most down-regulated in DZNep-sensitive cell lines. Red, relatively high expression; green, relatively low expression. (**B**) Quantitative levels of differentially expressed *ALOX5*. The *ALOX5* was determined by quantitative RT-PCR. Colunms, mean; bars, SE. Average C_T_ values were first normalized against the housekeeping gene β-*Actin* and converted to the induced fold change relative to the vehicle control.

Two sensitive MM cell lines (MM.1S and NCI-H929) and two relatively insensitive MM cell lines (KMS18 and OPM-2) were profiled on cDNA microarrays after exposure to vehicle control or DZNep. Many more transcripts were differentially expressed in response to DZNep in sensitive cell lines than in insensitive cell lines (**Fig. S1A, Tables S2, S3, S4**). Seventy-three genes were significantly up-regulated and 113 genes were significantly down-regulated in both of the sensitive cell lines. Gene ontology analysis revealed that these genes were remarkably enriched for their roles in cellular metabolism (**Fig. S1B**), suggesting cellular metabolism may play a vital role in MM cell sensitivity to DZNep induced apoptosis. The arachidonate 5-lipoxygenase (*ALOX5*) was among the genes most down-regulated by DZNep treatment (**Fig. 2A**). *ALOX5* has previously been linked to leukemia stem cells and cancer-related signaling pathways [17], [18], [19], [20], [21]. The differential expression of *ALOX5* indicates a potential role in determining sensitivity to DZNep in MM. Differential expression of *ALOX5* was confirmed by quantitative RT-PCR (**Fig. 2B**). The magnitude of decreased expression of *ALOX5* is similar in MM.1S and NCI-H929, but baseline levels of *ALOX5*expression are higher in NCI-H929 compared with MM.1S.

### DZNep reduces EZH2 and ALOX5 levels, and induces caspase-dependent cell apoptosis

**Figure 3:**
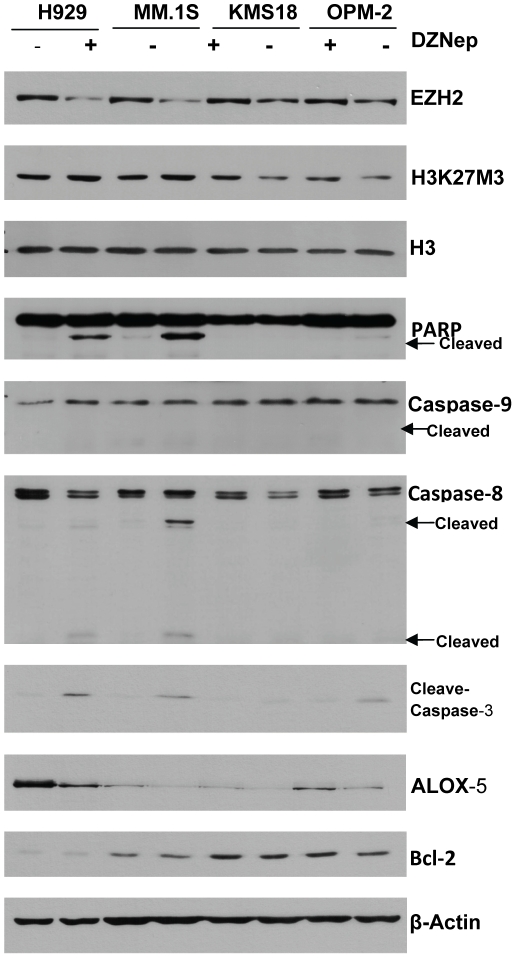
Analysis of DZNep effects by Western-blot and RT-PCR. DZNep reduced EZH2 and ALOX5 levels, and induced caspase-dependent cell apoptosis. Cells were treated with 0.5 umol/L DZNep for 72 h, and then whole-cell lysates were analyzed by Western-blot analysis. β-Actin was used as an internal control. Treatment with DZNep reduced protein levels of EZH2 in MM cells. ALOX5 was over-expressed in H929 and was down-regulated greatly by DZNep treatment. In H929 and MM1.S, DZNep efficiently induced caspase-3 activation and the cleavage of PARP. Furthermore, DZNep treatment induced caspase-8 activation and had little effect on full-length caspase-9 levels.

DZNep treatment has been demonstrated to decrease levels of EZH2 in solid tumors and acute myeloid leukemia[6], [7]. Consistent with these reports, treatment with DZNep also reduced protein levels of EZH2 in NCI-H929, MM.1S, KMS18 and OPM-2 (**Fig. 3** **A**). H3K27Me3 levels were also decreased in KMS18 and OPM-2 but not in the two sensitive cell lines, suggesting that the induction of cell death by DZNep in myeloma cells is not mediated through its effect on EZH2 or H3K27Me3. This result also suggests that proteins other than EZH2 may be responsible for the H3K27 trimethylation. Consistent with real-time PCR results, ALOX5 was over-expressed in NCI-H929 and decreased greatly by DZNep treatment on western blot. The other 3 cell lines had low levels of ALOX5 which was also decreased by DZNep.

To understand the mechanisms of DZNep-induced cell death in MM cells, we examined the involvement of caspase in cell death. In NCI-H929 and MM.1S, DZNep efficiently induced caspase-3 activation and the cleavage of PARP, a substrate of caspase-3. Furthermore, DZNep treatment induced caspase-8 activation and had little effect on caspase-9 activation, suggesting DZNep might induce apoptosis via the death receptor signaling pathway.

### Sensitivity to DZNep is modulated by expression levels of ALOX5

**Figure 4:**
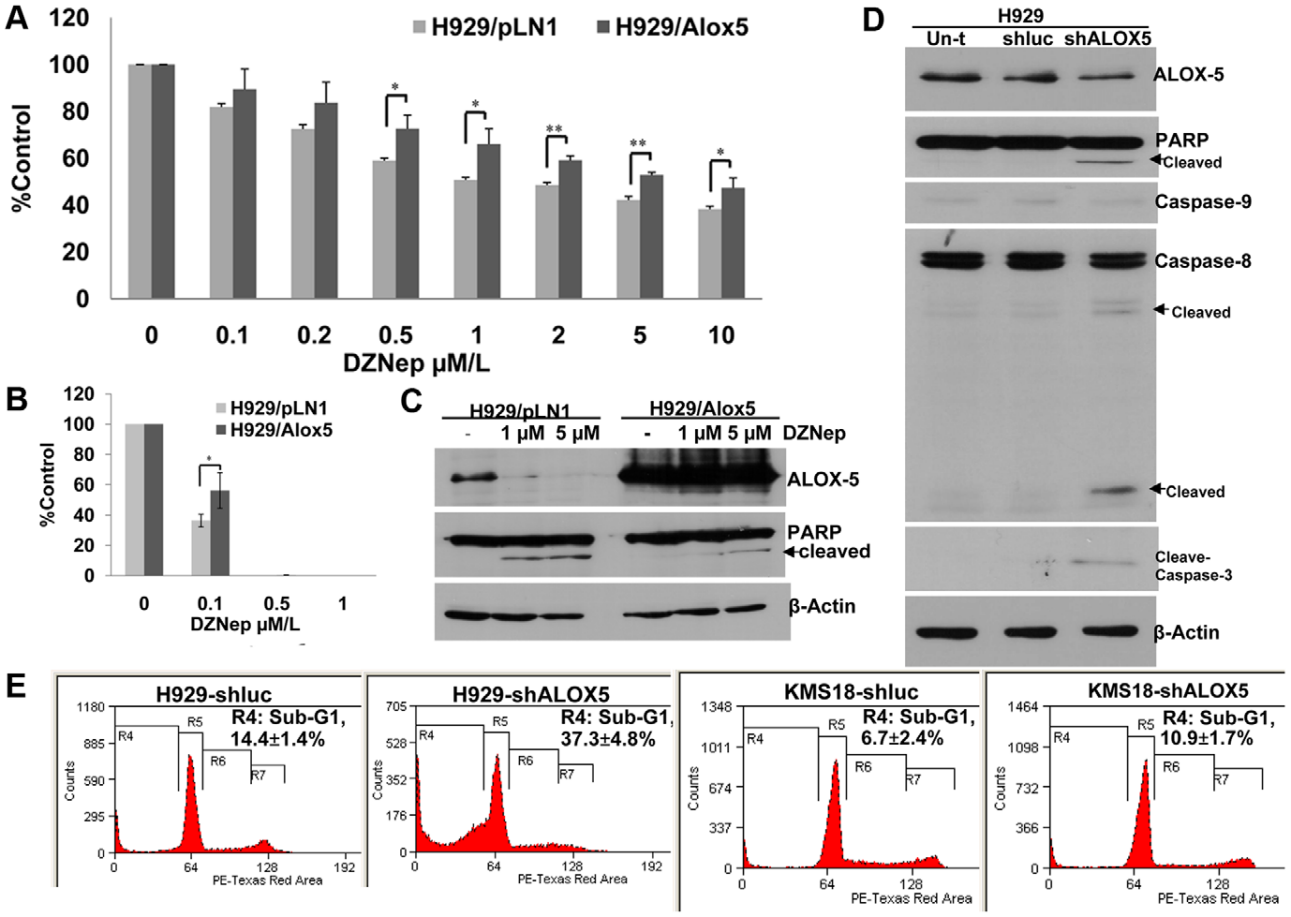
Modulation of ALOX5 expression affects sensitivity to DZNep in NCI-H929 cells. **()A** NCI-H929 cells expressing the ectopic ALOX5 were treated with DZNep for 72 h. The proliferation was determined using the MTS colorimetric assay. * indicates p<0.05, and ** indicates p<0.01. **(B)** Colony formation assay. Cells were treated with DZNep and cultured in methylcellulose media for 14 days. Average colony counts are presented. Data represent the mean ± SD derived from 3 separate experiments with duplicate wells per condition. **(C)** Western-blot analysis. Cells were also infected with shRNA control or shALOX5. **(D)** After 72 h, total cell lysates were prepared and Western-blot analysis was performed. **(E)** Apoptosis was measured by flow cytometric sub-G1 analysis.The shALOX5 treatment induced apoptosis in NCI-H929, while it had little effect on DZNep resistance in KMS18.

To investigate whether ALOX5 is directly associated with DZNep induced apoptosis, we generated the cell line of NCI-H929/ALOX5 overexpressing ALOX5. The proliferation assay indicated that overexpression of ALOX5 reduced sensitivity to DZNep and resulted in 3.2-fold increase of IC_50_ compared with vector control (**Fig. 4A**). To assay long-term effect of DZNep, we cultured control and NCI-H929/ALOX5 cells in methylcellulose with DZNep. NCI-H929/ALOX5 cells exhibited resistance to colony formation inhibition relative to control (**Fig. 4B** **and Fig. S3A and S3B**). Western-blot analysis showed a marked increase in the expression levels of ALOX5 in NCI-H929/ALOX5, and high levels of ectopic ALOX5 reduced PARP cleavage upon DZNep treatment (**Fig. 4C**). These data indicated that forced ectopic expression of ALOX5 could reduce sensitivity to DZNep.

To determine the effect of ALOX5 downregulation in cell viability, NCI-H929 was infected with the shALOX5. The shALOX5 mediated a significant decrease in ALOX5 mRNA and protein levels in NCI-H929 relative to a control shLuc (**Fig. S2 and** **Fig. 4D**). Similar to effect of DZNep, the shALOX5 also induced activation of caspase-8 and caspase-3 and cleavage of PARP, and had little effect on caspase-9 activation (**Fig. 4D**). Flow cytometric analysis indicated a significant increase of apoptosis after shALOX5 treatment in NCI-H929, while a minor increase in apoptosis was observed in control cell line KMS18 (**Fig. 4E**). Taken together, these data suggest that the downregulation of ALOX5 may contribute to cell viability of NCI-H929, and have the functional significance as a mediator of DZNep death response.

### *ALOX5* is aberrantly expressed in a subset of MM Patients

**Figure 5:**
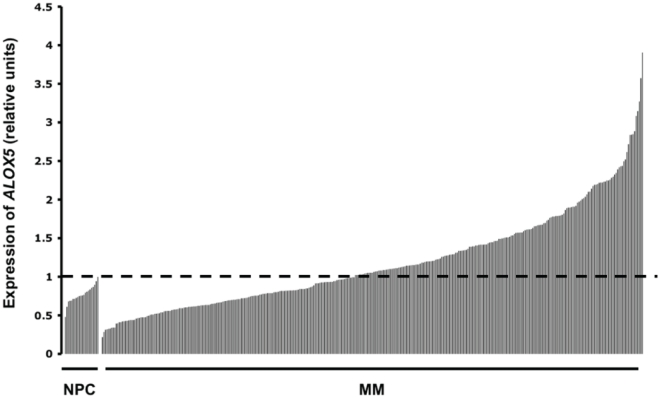
Expression of *ALOX5* in MM Patients. Raw expression value was obtained from GEO GSE 2658 and 5900. The expression values for probes representing *ALOX5* on the U133plus 2.0 chip were first log-transformed and median centered, and then a composite relative expression value for *ALOX5* for each patient was obtained by taking the median expression across these probes. This relative expression value is represented on the Y axis. The expression value of 22 NPC and 351 newly diagnosed MM are highlighted ranked from low to high expression. The horizontal dotted line represents the mean +2SD expression value among the NPC.

As DZNep induce apoptosis in myeloma cells in large parts through attenuating elevated expression of ALOX5, we wanted to see if *ALOX5* is also aberrantly expressed in human MM. We assessed a large gene expression dataset from the University of Arkansas Medical School that is available through the Gene Expression Omnibus (GSE2658 and GSE5900), and found that *ALOX5* expression was significantly higher in MM compared to NPC (*p*-value  = 0.002). Taking mean+2SD as the upper limit of normal expression, 187 of 351 (53%) MM patients have abnormal elevated expression of *ALOX5* **(** **Fig. 5** **)**. This suggests that our finding in the myeloma cell lines is potentially applicable to a subset of MM patients.

### Cotreatment with DZNep and ABT-737 synergistically inhibits growth and induces apoptosis of DZNep insensitive MM cells

**Figure 6:**
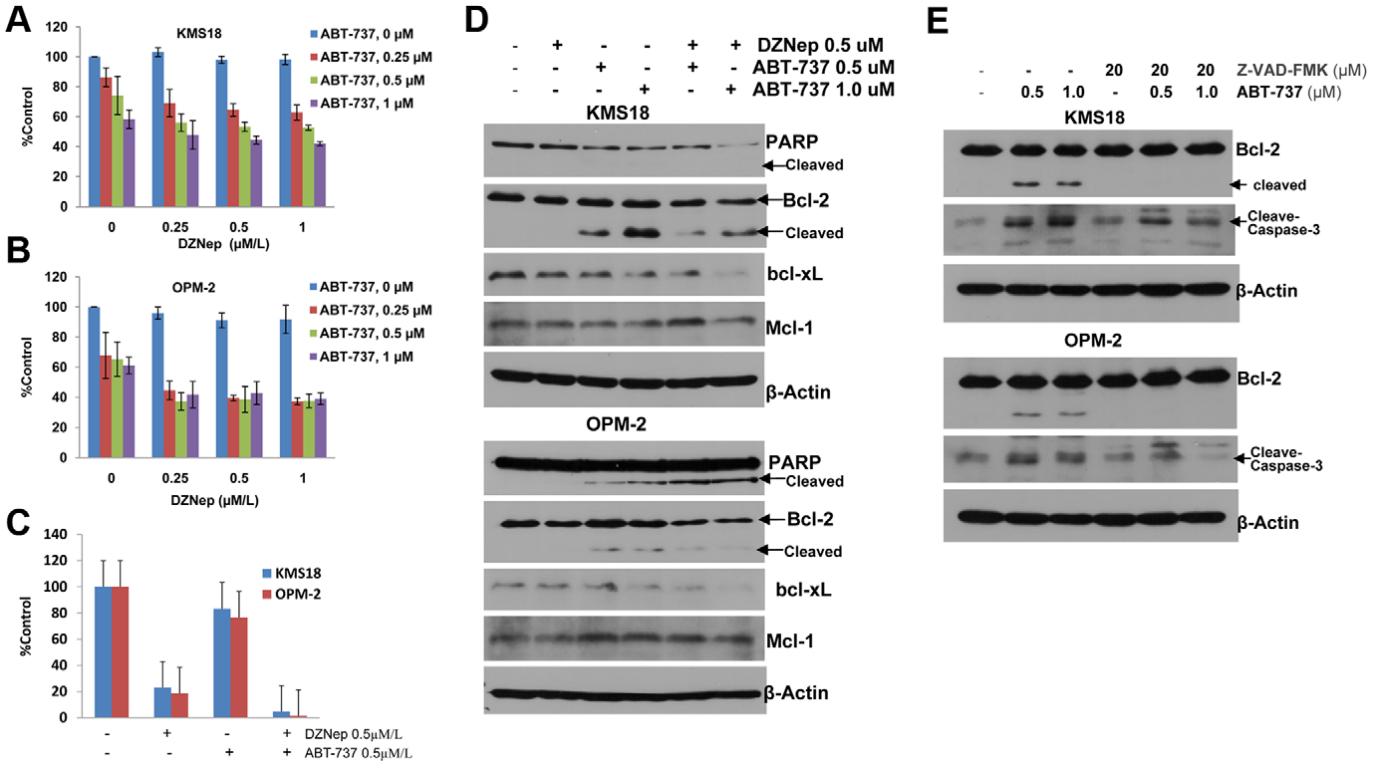
Cotreatment with DZNep and ABT-737 synergistically inhibits growth and induces apoptosis of DZNep insensitive MM cells. **(A)** Analysis of dose-effect relationship for DZNep (500 nmol/L) and ABT-737 (500 or 1000 nmol/L) for the proliferation after 72 h of exposure in MM cells. The proliferation was determined by MTS colorimetric assay. Data represent the mean ± SD derived from 3 separate experiments with triplicate wells per condition. After this, the CI values were calculated. CI <1, CI  = 1, and CI >1 represent synergy, additivity, and antagonism of the 2 agents, respectively. **(B)** Colony formation assay. Data represent the mean ± SD derived from 3 separate experiments with duplicate wells per condition. **(C)** After 72 h treatments, total cell lysates were prepared and Western-blot analysis was performed. **(D)** After treatment with ABT-737 for 48 h, Bcl-2 levels were determined by Western-blot. The Bcl-2 cleavage was inhibited by 1 h of preincubation with a pan-caspase inhibitor Z-VAD-FMK (Promega).

Bcl-2 is widely expressed in MM cell lines and primary patient samples and is correlated with resistance to chemotherapeutics [22], [23], [24], [25]. We hypothesize that Bcl-2 may also be associated with DZNep insensitivity in MM. As expected, elevated expression of Bcl-2 was detected in KMS18 and OPM-2 (**Fig. 3**). To see if inhibition of Bcl-2 can re-sensitize the resistant cells to DZNep, we determined the combined effects of DZNep and ABT-737, a Bcl-2 inhibitor, in MM cells. MTS assay demonstrated that, compared with each agent alone, combined treatment with DZNep and ABT-737 synergistically inhibited growth of KMS18 (DZNep at 0.25 or 0.5 µM/L) and OPM-2 cells, as indicated by combination indices of less than 1.0 (**Fig. 6A, 6B** **, and** **Table 1**). Colony formation assay indicated that KMS18 and OPM-2 cells exhibited a striking loss of colony formation upon cotreatment with DZNep and ABT-737 (**Fig. 6C** **and Fig. S3C and S3D)**. In addition, cotreatment with DZNep and ABT-737 decreased Bcl-2 and bcl-xL levels, and induced more PARP cleavage in KMS18 and OPM-2 cells (**Fig. 6D**). ABT-737 treatment had little effect on Mcl-1 levels. This is consistent with a previous study that showed high affinity binding of ABT-737 to Bcl-2 and bcl-xL, but not to the less homologous protein Mcl-1 [26]. In accordance with the synergistic effect of ABT-737 with DZNep, Bcl-2 shRNA treatment could also increase sensitivity to DZNep **(Fig. S4)**,

**Table 1:** The combination index (CI) for DZNep and ABT-737 combination.

DZNep (µM/L)	ABT-737 (µM/L)	I/KMS18	I/OPM-2
0.25	0.25	0.63	0.013
0.25	0.5	0.694	0.006
0.25	1	0.909	0.026
0.5	0.25	0.797	0.007
0.5	0.5	0.876	0.01
0.5	1	1.048	0.034
1	0.25	1.245	0.008
1	0.5	1.322	0.011
1	1	1.432	0.021

Note; A CI of less than, equal to, and more than 1 indicates synergy, additivity, and antagonism, respectively.

Previous reports showed ABT-737 disrupted an intracellular Bcl-2 family protein–protein interaction [26], while our western-blot analysis indicated ABT-737 could also induce Bcl-2 cleavage, represented by a small fragment recognized by Bcl-2 antibody. As Bcl-2 could be cleaved by caspases and cleavage of Bcl-2 could further activate downstream caspases [27], [28], and ABT-737 could induce activation of caspases in MM cell lines [29], [30], we examined whether ABT-737 induced Bcl-2 cleavage by a caspase-dependent pathway. As expected, cleavage of Bcl-2 induced by ABT-737 could be inhibited by pre-incubation with a pan-caspase inhibitor Z-VAD-FMK (**Fig. 6E**).

## Discussion

DZNep was previously reported to be a selective inhibitor of H3K27 and H4K20 trimethylation [6]. The extended study showed that DZNep globally inhibited histone methylation and was not selective [31]. Because there are multiple epigenetic mechanisms that deregulate gene expression during carcinogenesis, a molecule such as DZNep that inhibits multiple marks may be useful in epigenetic therapy.

MM is an incurable hematologic malignancy characterized by recurrent chromosomal translocations [32], [33]. MMSET (multiple myeloma SET domain) was identified as a gene involved in the t(4;14)(p16;q32) translocation affecting approximately 15% patients [34], [35]. This subtype of myeloma has a poor prognosis with frequent relapse after autologous stem-cell transplantation [34], [36], [37]. MMSET contains a SET domain that is found in many histone methyltransferases and determines their enzymatic activity[38]. MMSET has been shown to be a transcriptional corepressor and increase H4K20 trimethylation [39], so we hypothesized that DZNep should be useful for epigenetic therapy against MM with t(4;14). However, our proliferation and apoptosis assays indicated DZNep sensitivity was not associated with t(4;14) in MM (**Table S5**). In addition, the sensitivity of myeloma cells to DZNep is not directly related to inhibition of EZH2 or H3K27 trimethylation, suggesting the DZNep may also act through non-epigenetic mechanisms. The precise mechanism of drug activity in myeloma remains elusive and is being actively pursued by our group.

Nevertheless, insights into the determinants of DZNep sensitivity was derived from gene expression array analysis of MM cells treated with vehicle control or DZNep. Transcripts differentially expressed in response to DZNep in MM were remarkably enriched for their roles in cellular metabolism, suggesting cellular metabolism might play a vital role in MM cell sensitivity to DZNep induced apoptosis. Previous studies showed that metabolic pathways are not only involved in the proliferation of cancer cells, but also regulated their apoptosis [40]. *ALOX5*, the most down-regulated gene by DZNep in sensitive MM cells, is a member of the lipoxygenase gene family and plays an essential role in the biosynthesis of leukotrienes from arachidonic acid [41]. Several groups have examined the role of ALOX5 pathway in carcinogenesis and have shown that it plays an important role in promoting tumor growth and survival [17], [19], [20], [21], [42]. Recently it was shown that the loss of *ALOX5* gene impaired leukemia stem cells and prevented chronic myeloid leukemia in mice [18]. These prompted us to determine whether overexpression of ALOX5 is associated with DZNep sensitivity. Our studies indicated DZNep treatment could decrease ALOX5 level, and ectopic expression of ALOX5 reduced sensitivity to DZNep in NCI-H929. Furthermore, knocking down *ALOX5* could also induce apoptosis with activation of caspases. These results suggest that ALOX5 has the functional significance as a mediator of DZNep induced apoptosis. However, ectopic expression of ALOX5 incompletely prevents NCI-H929 cells from DZNep induced apoptosis, so the apoptosis might depend on the change of multiple proteins or signaling pathways. Several striking differences such as *FADS1* and *SCD5* involved in cellular metabolism were also observed in DZNep sensitive cell lines in addition to the changes in *ALOX5* (**Table S2)**.

The overexpression of Bcl-2 is almost universal in MM cell lines and primary patient samples and promotes inappropriate survival leading to resistance to therapy with interferon, dexamethasone, etoposide, doxorubicin and bortezomib[22], [23], [24], [25]. ABT-737 is a small-molecule inhibitor designed to specifically inhibit antiapoptotic proteins of the Bcl-2 family. This molecule is a BH3 (Bcl-2 homology region 3) mimetic that binds with high affinity to Bcl-xL, Bcl-2 and Bcl-w. Binding of ABT-737 to a hydrophobic groove on Bcl-2 family proteins prevents binding of Bax, tilting the balance of pro- and antiapoptotic molecules in favor of cellular apoptosis [26], [29]. Previous studies reveal that ABT-737 synergizes with a range of cytotoxic chemotherapy agents [26], [43], [44]. We found that Bcl-2 was overexpressed in DZNep insensitive cell lines and combined treatment with DZNep and ABT-737 synergistically induced apoptosis in these cells. This also suggested that overexpression of Bcl-2 might be associated with DZNep insensitivity. Although ABT-737 inhibits antiapoptotic functions of Bcl-2 family through competing with BAD for docking to the hydrophobic groove of Bcl-2/Bcl-xL, thus increasing activity of pro-apoptotic BH3 proteins, we found that ABT-737 could also promote Bcl-2 cleavage, represented by a small fragment recognized by Bcl-2 antibody through a caspase dependent pathway.

In conclusion, this study showed that one of the determinants of DZNep efficacy in MM is the ability to down-regulate elevated ALOX5 levels. As ALOX5 is over-expressed in a substantial subset of MM patients, this may be is viable therapeutic strategy in a subset of MM. In addition, DZNep insensitivity might be associated with the overexpression of Bcl-2, and the combination of ABT-737 and DZNep could synergistically induced apoptosis in DZNep insensitive MM.

## Supporting Information

Figure S1 **The cDNA microarray analysis of gene expression changes in response to DZNep treatment.** **(A)** The column diagram showing the numbers of differentially expressed genes in DZNep-treated cell lines. Much more number of transcripts were differentially expressed in response to DZNep in sensitive cell lines than in insensitive cell lines. (**B**) Gene ontology (GO) assignments of biological process of genes. GO analysis revealed that these genes were remarkably enriched for their roles in cellular metabolism.
(DOCX)

Figure S2 **NCI-H929 cells were infected with shRNA control or shALOX5.** After 48 h, *ALOX5* mRNA level was analyzed by quantitative RT-PCR.
(DOCX)

Figure S3 **Colony formation assay in methylcellulose media**. Cells were plated in duplicate at a density of 1,500 or 3,000 cells/mL in 0.4 mL volume in 24-well plates. Plates were incubated at 37°C, 5% CO_2_, and ≥95% humidity for 14 days. To observe colonies, cells were stained with 0.5 mg/mL metabolizable tetrazolium salt. After incubating at 37°C, 5% CO_2_ for 1 h, images were acquired at 40X magnification with an Olimpus IX71 inverted microscope. Each experiment was performed 3 times, and representative examples are shown. **(A) and (B)** Ectopic expression of ALOX5 reduced sensitivity to DZNep. **(C) and (D)** Cotreatment with DZNep and ABT-737 synergistically reduced colony formation in KMS18 and OPM-2.
(DOCX)

Figure S4 **Potentiation effect of Bcl-2 shRNA with DZNep in KMS18 and OPM-2 MM cells.** Cells were treated with Bcl-2 shRNAs and cultured for 3 days with or without DZNep treatment. Luc, control shRNA; #82 and #83, Bcl-2 shRNA. **(A) and (B)** Bcl-2 shRNAs increased sensitivity to DZNep. The proliferation was determined by MTS colorimetric assay (Promega). Data represent the mean ± SD derived from 3 separate experiments with triplicate wells per condition. **(C)** Quantitative RT-PCR analysis of Bcl-2 mRNA level on 48 h of Bcl-2 shRNA treatment. Colunms, mean; bars, SE. Average C_T_ values were first normalized against the housekeeping gene β-*Actin* and converted to the induced fold change relative to the vehicle control. **(D)** Western-blot analysis of apoptosis and Bcl-2 expression.
(DOCX)

Table S1 **Primer Sequences for quantitative PCR.**
(XLSX)

Table S2 **Specially differentially expressed genes (fold changes >2) in sensitive MM cell lines (H929 and MM1.S) exposed to DZNep.**
(XLSX)

Table S3 **Specially differentially expressed genes (fold changes >2) in insensitive MM cell lines (KMS18 and OPM-2) exposed to DZNep.**
(XLSX)

Table S4 **Common differentially expressed genes (fold changes >2) in MM cell lines exposed to DZNep.**
(XLSX)

Table S5 **Sensitivity to DZNep wasn't associated with t(4;14) translocation in MM.**
(XLSX)
