# Supplementary material for: Determinants of Sensitivity to DZNep Induced Apoptosis in Multiple Myeloma Cells
Source: PLoS One. 2011 Jun 24;6(6):e21583. doi: 10.1371/journal.pone.0021583 (PMC3123372; doi:10.1371/journal.pone.0021583)
Supplement: Figure S1 — The cDNA microarray analysis of gene expression changes in response to DZNep treatment. (A) The column diagram showing the numbers of differentially expressed genes in DZNep-treated cell lines. Much more number of transcripts were differentially expressed in response to DZNep in sensitive cell lines than in insensitive cell lines. (B) Gene ontology (GO) assignments of biological process of genes. GO analysis revealed that these genes were remarkably enriched for their roles in cellular metabolism. (DOCX) [file pone.0021583.s001.docx]

**Fig. S1A**

**Fig. S1B**
