# Supplementary figures and images for: Determinants of Sensitivity to DZNep Induced Apoptosis in Multiple Myeloma Cells
Source: PLoS One. 2011 Jun 24;6(6):e21583. doi: 10.1371/journal.pone.0021583 (PMC3123372; doi:10.1371/journal.pone.0021583)

**Fig. S2**

**
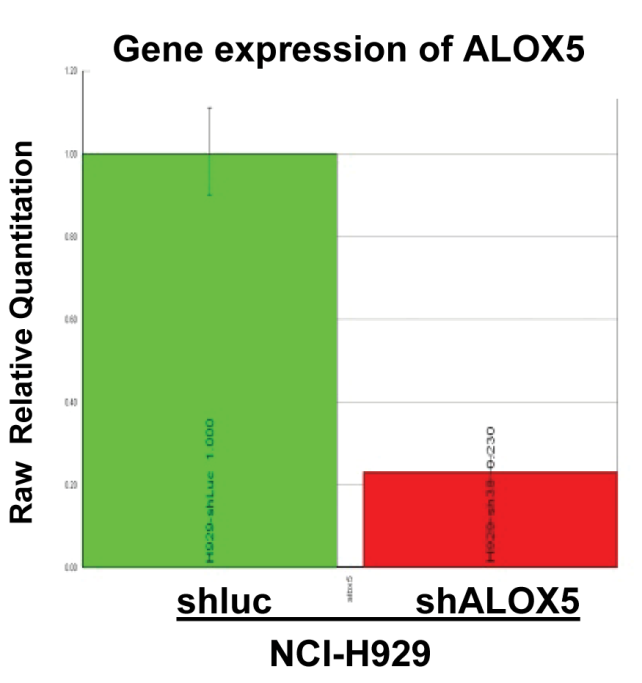
**

Supplement: Figure S2 — NCI-H929 cells were infected with shRNA control or shALOX5. After 48 h, ALOX5 mRNA level was analyzed by quantitative RT-PCR. (DOCX) [file pone.0021583.s002.docx]

**Fig. S4A**

**Fig. S4B**

**Fig. S4C**

**
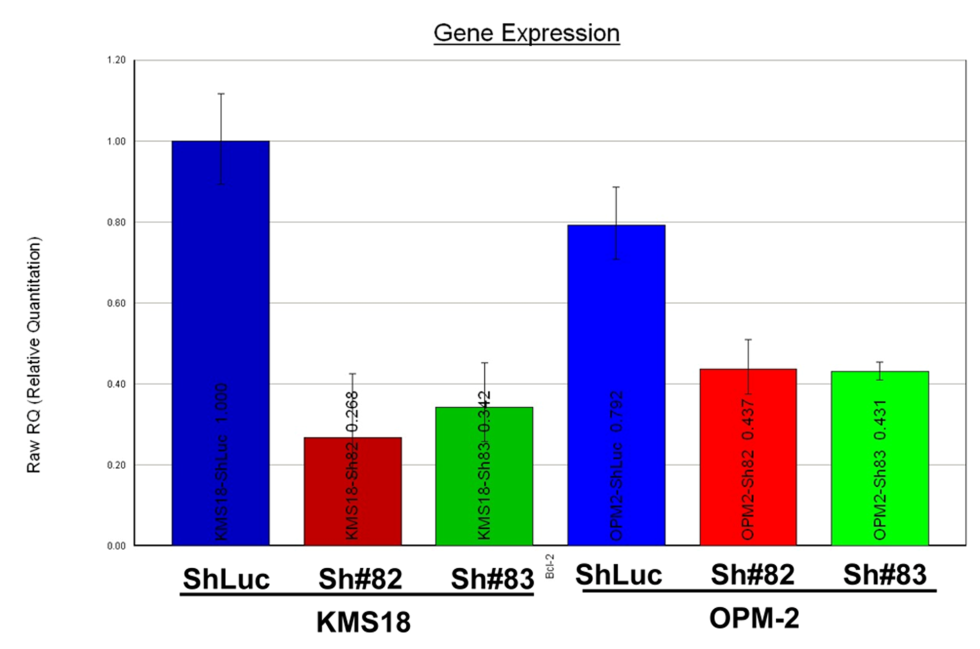
**

**Fig. S4D**

**
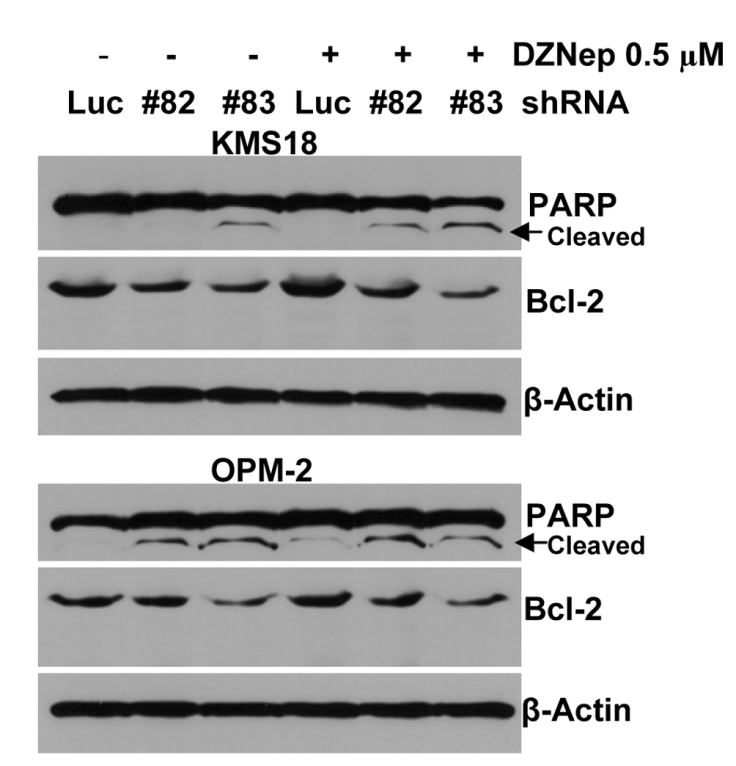
**

Supplement: Figure S4 — Potentiation effect of Bcl-2 shRNA with DZNep in KMS18 and OPM-2 MM cells. Cells were treated with Bcl-2 shRNAs and cultured for 3 days with or without DZNep treatment. Luc, control shRNA; #82 and #83, Bcl-2 shRNA. (A) and (B) Bcl-2 shRNAs increased sensitivity to DZNep. The proliferation was determined by MTS colorimetric assay (Promega). Data represent the mean ± SD derived from 3 separate experiments with triplicate wells per condition. (C) Quantitative RT-PCR analysis of Bcl-2 mRNA level on 48 h of Bcl-2 shRNA treatment. Colunms, mean; bars, SE. Average CT values were first normalized against the housekeeping gene β-Actin and converted to the induced fold change relative to the vehicle control. (D) Western-blot analysis of apoptosis and Bcl-2 expression. (DOCX) [file pone.0021583.s004.docx]
