# Supplementary material for: Determinants of Sensitivity to DZNep Induced Apoptosis in Multiple Myeloma Cells
Source: PLoS One. 2011 Jun 24;6(6):e21583. doi: 10.1371/journal.pone.0021583 (PMC3123372; doi:10.1371/journal.pone.0021583)
Supplement: Figure S3 — Colony formation assay in methylcellulose media. Cells were plated in duplicate at a density of 1,500 or 3,000 cells/mL in 0.4 mL volume in 24-well plates. Plates were incubated at 37°C, 5% CO2, and ≥95% humidity for 14 days. To observe colonies, cells were stained with 0.5 mg/mL metabolizable tetrazolium salt. After incubating at 37°C, 5% CO2 for 1 h, images were acquired at 40X magnification with an Olimpus IX71 inverted microscope. Each experiment was performed 3 times, and representative examples are shown. (A) and (B) Ectopic expression of ALOX5 reduced sensitivity to DZNep. (C) and (D) Cotreatment with DZNep and ABT-737 synergistically reduced colony formation in KMS18 and OPM-2. (DOCX) [file pone.0021583.s003.docx]

**Fig. S3A**

**H929/pLN1, DZNep 0.1 µM**

**H929/pLN1, DZNep Control**


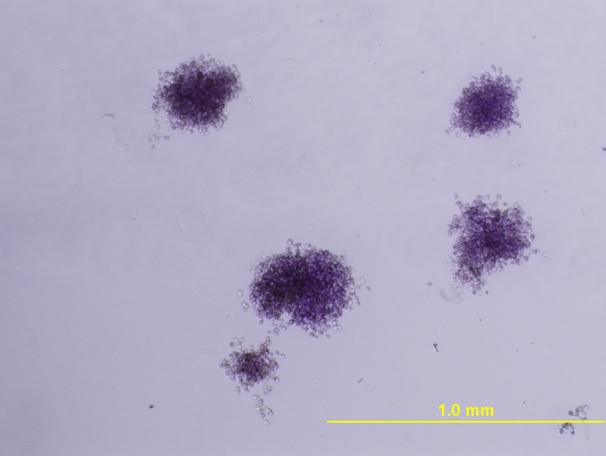

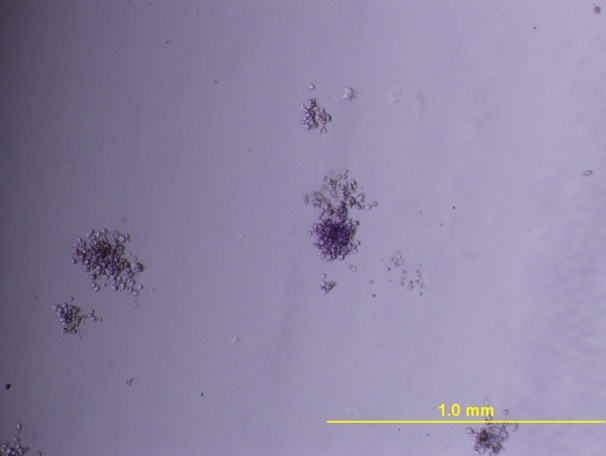


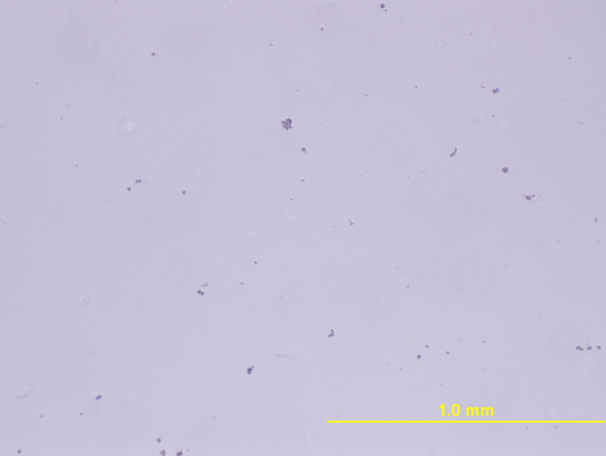

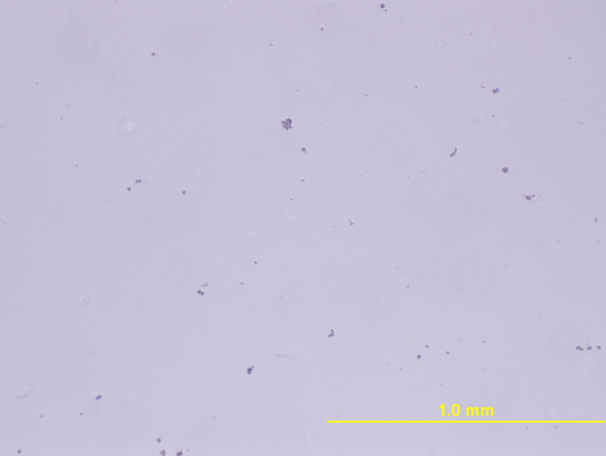


**H929/pLN1, DZNep 0.5 µM**

**H929/pLN1, DZNep 1 µM**

**Fig. S3B**

**H929/ALOX5, DZNep 0.1µM**

**H929/ALOX5, Control**


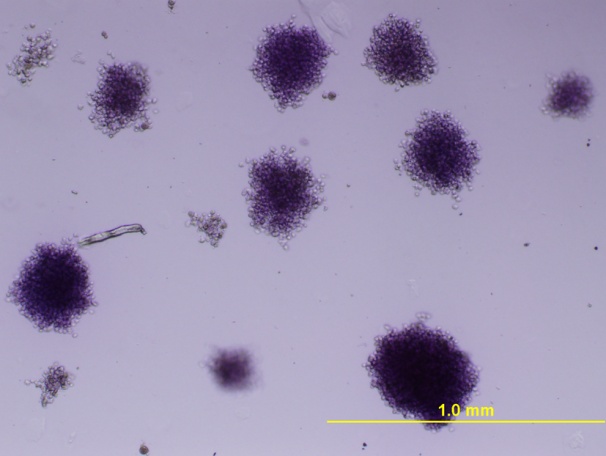

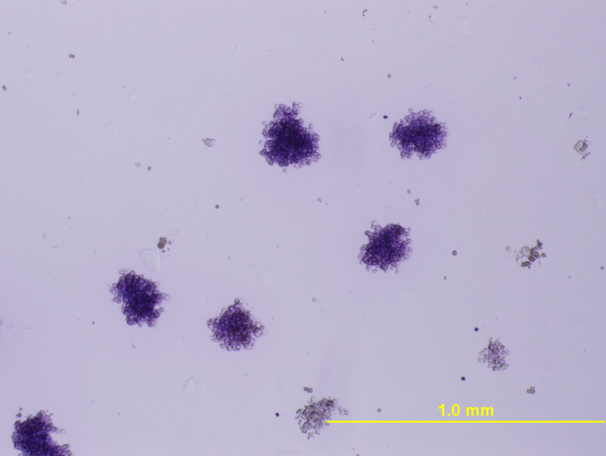


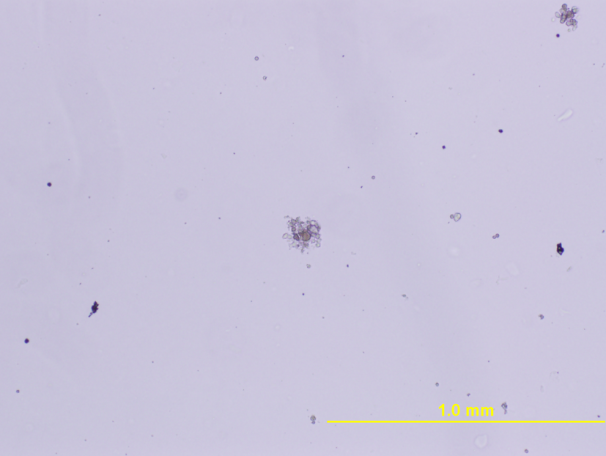

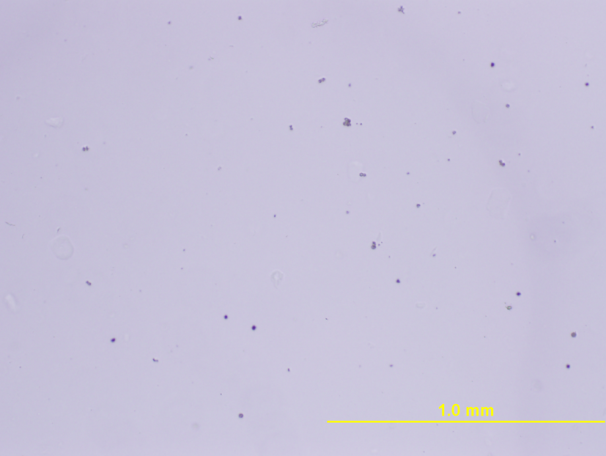


**H929/ALOX5, DZNep 0.5µM**

**H929/ALOX5, DZNep µM**

**Fig. S3C**


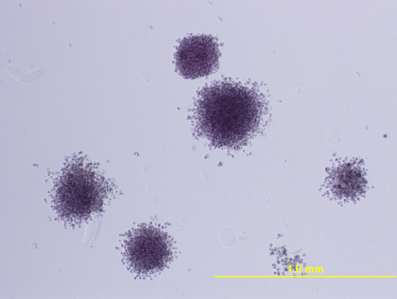

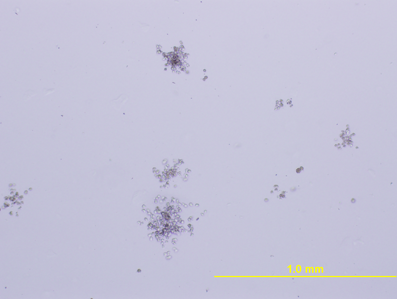


**KMS18, DZNep 0.5µM +ABT-737 0.5µM**

**KMS18, ABT-737 0.5µM**

**KMS18, DZNep 0.5µM**

**KMS18, Control**


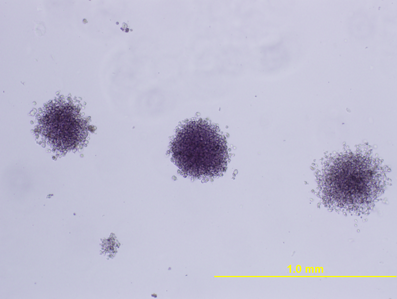

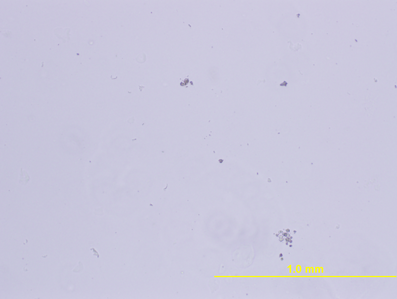


**Fig. S3D**

**OPM-2, DZNep 0.5µM**

**OPM-2, Control**


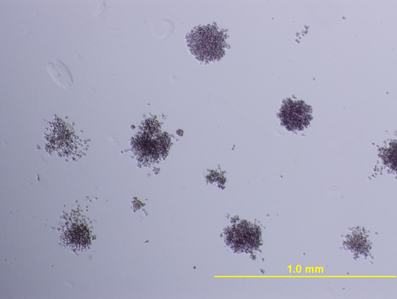

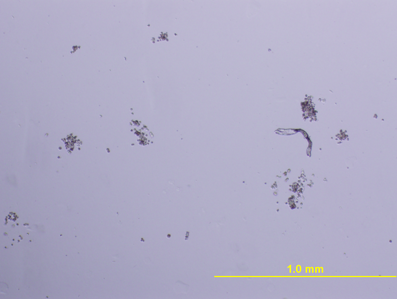


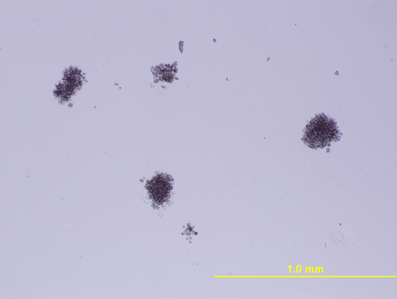

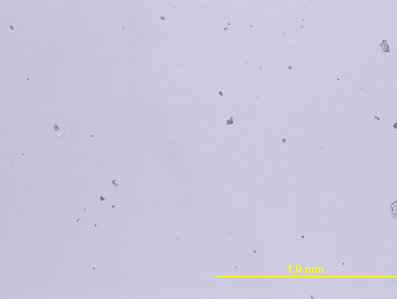


**OPM-2, DZNep 0.5µM +ABT-737 0.5µM**

**OPM-2, ABT-737 0.5µM**
